# Characterizing Adverse Events of Cranioplasty Implants After Craniectomy: A Retrospective Review of the Federal Manufacturer and User Facility Device Experience Database

**DOI:** 10.7759/cureus.16795

**Published:** 2021-07-31

**Authors:** Caitlin-Craft Hacherl, Neal A Patel, Keri Jones, Nikki B Ruh, Julian L Gendreau, Mickey E Abraham, Antonios Mammis

**Affiliations:** 1 Neurological Surgery, Mercer University School of Medicine, Macon, USA; 2 Neurological Surgery, Mercer University School of Medicine, Savannah, USA; 3 Medicine, Dwight D. Eisenhower Army Medical Center, Augusta, USA; 4 Medicine, Walter Reed National Military Medical Center, Bethesda, USA; 5 Biomedical Engineering, Johns Hopkins University, Baltimore, USA; 6 Neurological Surgery, University of California San Diego, San Diego, USA; 7 Neurological Surgery, New York University Grossman School of Medicine, New York, USA

**Keywords:** adverse events, complications, craniectomy, cranioplasty, maude, neurotrauma, plate

## Abstract

Introduction

Cranioplasty is performed by placing an artificial plate in place of a patient's native skull bones to repair post-craniectomy defects after trauma. Implanted materials can range from titanium to synthetic polyether derivatives and are produced by multiple manufacturers. There are few studies characterizing complications associated with these cranioplasty plates to date. We aimed to quantify and categorize complications of these devices using a national federal database.

Methods

The Manufacturer and User Facility Device Experience (MAUDE) database was queried for all entries reported under the category "plate, cranioplasty, preformed, non-alterable" with the additional product code GXN between the time range from September 1, 2010, to September 1, 2020. After data extraction, each of the entries was screened for duplicates and tabulated into different categories of complications. Additionally, product information such as the plate manufacturer was extracted from each entry.

Results

The search yielded 329 unique event reports. The most frequent complications were infection (39%), followed by incorrectly fitting implants (30%) and implant breaks (6%). Other major complications included cerebrospinal fluid leakage and cerebral edema (5%), wound dehiscence (5%), and migration of hardware (3%). The brands associated with the most entries in the database were the Synthes (DePuy Synthes Companies, Massachusetts, United States) polyetheretherketone (PEEK) patient-specific implants (PSI) (57%), the Biomet (Zimmer Biome, Indiana, United States) hard tissue replacement-polyetherketoneketone (HTR-PEKK) patient-matched implant (PMI) (23%), and the AccuShape PEEK PSI (MedCAD, Dallas, USA) (5%).

Conclusions

Infection and improperly fitting implants appear to be the two most frequent complications of cranioplasty plates. The goals of future research should include the prevention of plate infections as well as improving techniques to custom-fit implantable devices.

## Introduction

Cranioplasties are performed after craniectomy surgeries for the purpose of cosmetically preserving the cranial shape, maintaining proper cerebrospinal fluid flow, maintaining adequate blood flow, and preventing direct atmospheric pressure from damaging the unprotected brain [1]. Neurosurgeons have the option of performing cranioplasties either by replacing a patient’s native bone or by placing artificial hardware if the patient’s original skull is not suitable for replacement.

These artificial implant materials have evolved over the years and have included the use of titanium mesh, polyethylene polymers, methyl methacrylate, calcium phosphate cement, and hydroxyapatite-polymethyl methacrylate composite [2,3]. Multiple manufacturers create and distribute implantable plates and clamps used for cranioplasties, leaving a lack of consensus on which material formulation is the best [4-6]. Additionally, in the early 2000s, polyetheretherketone (PEEK) was newly incorporated into the variety of implant materials available for use in cranioplasties [3,4].

Even with this wide variety of options of inserts for cranioplasty surgeons, there has been little research performed describing complications of artificial cranioplasty plates after surgery [3,7-9]. Therefore, this study aimed to provide a further assessment of post-operative complications of artificial cranioplasty plates after surgery by characterizing complications reported to a large federal database. To accomplish this aim, the federal Manufacturer and User Facility Device Experience (MAUDE) database was queried for all complication entries reported for non-alterable cranioplasty plates during the past 10 years.

## Materials and methods

The MAUDE database was created and maintained by the United States Food & Drug Administration (FDA) for receiving and organizing adverse event reports of any approved medical device in the United States [10]. The information contained within these reports is used for the purpose of post-hoc risk assessment after devices are approved via original clinical trials and FDA. Medical device reports (MDRs) are completed by device manufacturers, physicians, and facilities at which the device is used.

The MAUDE database was initially searched using the keywords “plate, cranioplasty, preformed, non-alterable,” while using product code GXN. This search yielded 485 results. The reporting period of September 1, 2010, to September 1, 2020, was queried.

Reports were first stratified by date to identify any duplicates to be later removed when reviewing. Reports filed on the same date were flagged for further investigation as possible duplicate entries. The potential for additional duplicate entries was assessed by comparing entry descriptions. Reports for complications of cranioplasty clamps were excluded from the analysis. There were eight entries within the MAUDE raw data that did not pertain to cranioplasties, which were found when categorizing the events and were not included in the final count of unique adverse events.

The authors obtained all extracted data and formulated appropriate categories used for this assessment. Next, entries from the database were stratified into these categories. The data was analyzed and visualized with summations and percentages using descriptive statistics within Microsoft Excel (Microsoft Corporation, Washington, United States) program for complication categories. Event categorization was determined based on the primary complaint, i.e., if a patient-specific Implant (PSI) did not fit correctly and subsequently broke as a result, then the event would be classified as an incorrect fit.

After the initial categorization, several subcategorizations were made. Infections were subdivided into different types and corresponding frequencies. In addition, it was further stratified by reported infectious organisms. Subsequent action taken after the complications were also recorded for each entry that reported subsequent action. Finally, each adverse event was also categorized by the brand of implant that had the reported complication.

## Results

**Figure 1:**
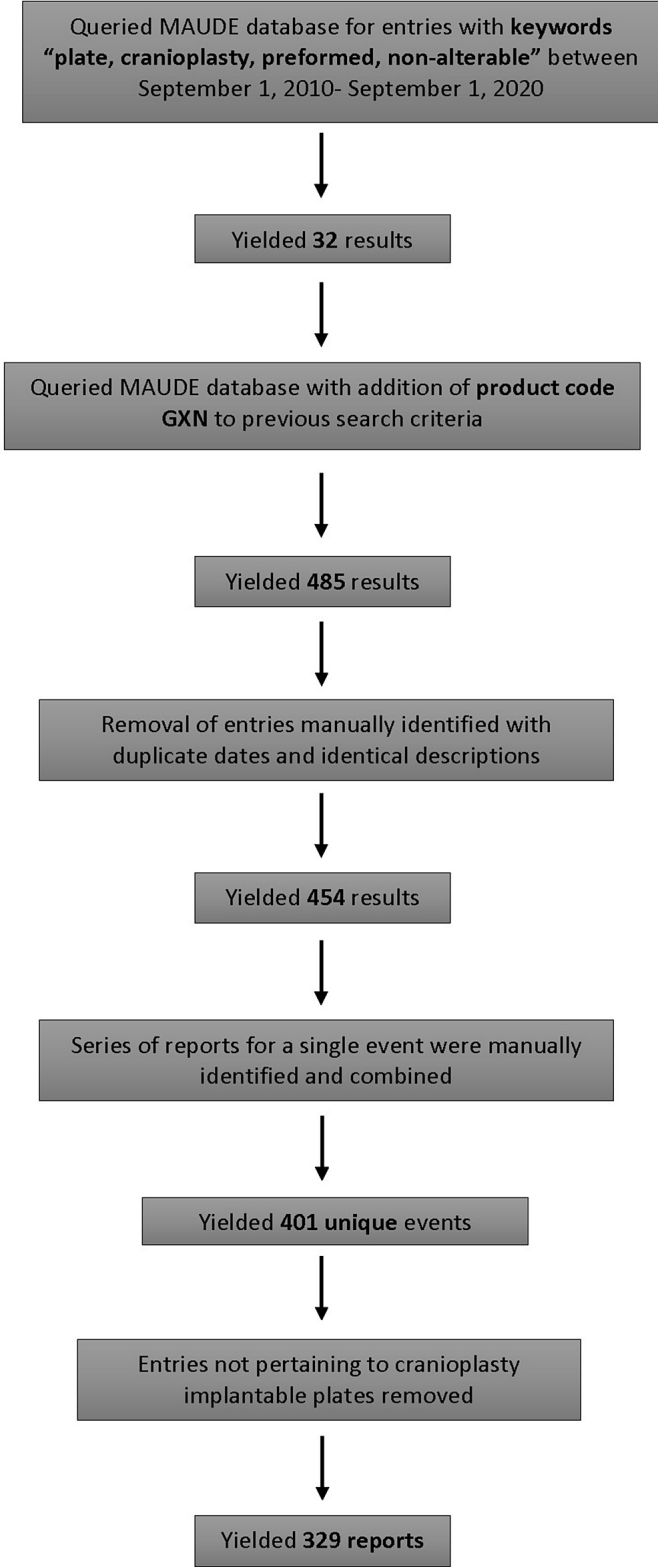
Flowsheet depicting stages of the database search to the eventual selection of included reports

The original database search retrieved a total of 485 entries. After duplicate entries, reports for clamps and reports that did not pertain to cranioplasty plates were removed from the dataset, and 329 unique event reports remained. The data search and duplicate removal process are outlined as a flowsheet in Figure 1.

Overall, the most frequent complications were infection (39%), followed by incorrect size or brand of the implant (30%), and the physical breaks of equipment or assembly issues (6%). Other complications included wound dehiscence and problems with wound healing (5%) and cerebrospinal fluid (CSF) leaks, cerebral edema, increased intracranial pressure (ICP), and epidural fluid collection (5%). Less frequent adverse events included device migration and unintended movement (3%), complications due to patient conditions (2%), and other classifications (4%). There were two reported incidences of focal neurological deficits, one reported seizure, and two reports of pain experienced by the patient. Five cases of cutaneous and allergic reactions were reported, and there were three reports of radiation-induced implant exposure or malfunction. Complications of excessive post-operative bleeding and hematoma were each reported once. All complication categories as defined by the authors with the total number of corresponding event reports are listed in Table 1.

**Table 1:** Complication categories defined by authors and corresponding frequency of entries CSF, Cerebrospinal fluid; ICP, intracranial pressure.

Complication	Total
Infection	128
Incorrect size or brand of implant	100
Clamp, plate, lead break, or assembly issue	21
Wound healing problem, wound dehiscence	18
CSF leak, cerebral edema, epidural fluid collection, or increased ICP	18
Unknown, other	14
Device migration	9
Due to pre-existing patient condition	6
Allergic reaction	5
Focal neurological deficits, seizures, pain	5
Radiation-induced implant malfunction	3
Excessive post-operative bleeding, hematoma	2

**Figure 2:**
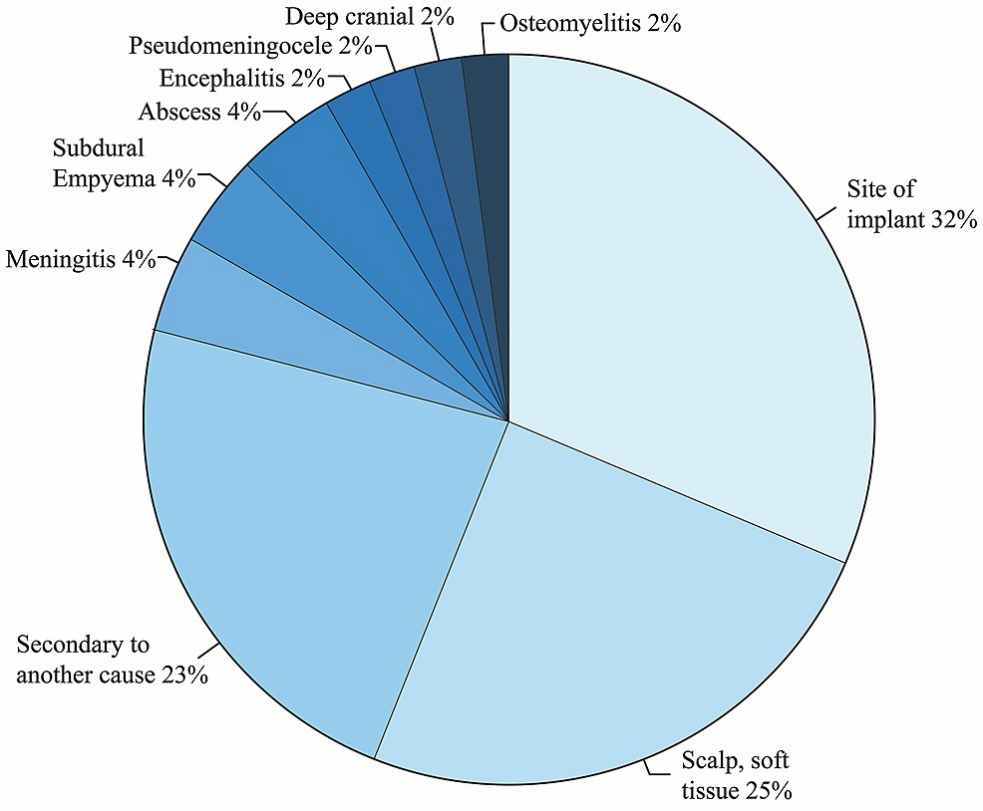
Infection types as specified in the event reports

Infections were subdivided by types, revealing that 33% occurred at the implant site, 24% were scalp or soft tissue-related infections, and 24% were secondary to another cause. The location or cause of infection was not specified for 62% of all cases. For infections occurring at the site of implant, three cases were caused by *Staphylococcus aureus*, two cases by *Propionibacterium acnes*, and one case by *Escherichia coli*. The causal organism for nine of the 15 implant site infections was not specified. Eleven percent of infections resulted in meningitis (two), abscesses (two), and subdural empyema (one). The final 8% of infections resulted in one reported complication of osteomyelitis, encephalitis, deep cranial infection, and a pseudomeningocele. The subdivision of infection types is illustrated in Figure 2.

Eighty-four percent of event reports were followed up with an explant of the device, replacement with a new implant, or revision surgery, leaving the original device in place. The remaining 16% of event reports did not specify what subsequent action was taken.

For infectious complications, 66% of plates were explanted, 18% of plates were replaced, and 9% of infected plates were left in place following revision. The majority (61%) of plates that were incorrectly sized were revised and left in place, but 15% were explanted, and 7% were replaced with new implants. After a plate broke, 33% of these devices were replaced, 29% were revised and left in place, and 14% were explanted; 24% of reports did not specify any subsequent management. Complications of wound healing and wound dehiscence were mostly managed with explant of plates (61%, n = 18); 28% of plates were replaced, but one plate was revised and left in place.

**Figure 3:**
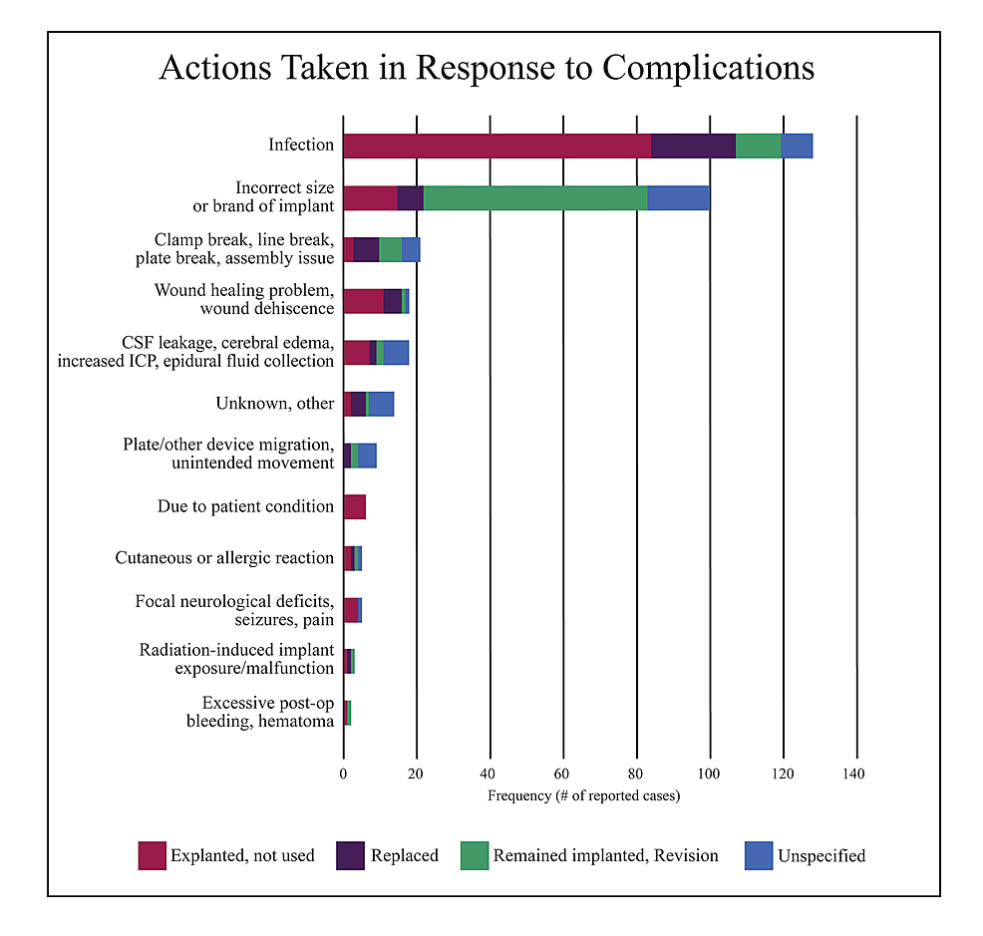
Frequency of subsequent action taken for each complication category CSF, Cerebrospinal fluid.

The majority of remaining complication categories including those related to allergic reactions, fluid leakage or collection, focal neurological deficits, plate migration, bleeding, and complications arising from pre-existing patient conditions were managed with the explant of the device. These complications were rarely managed by revision surgery. Figure 3 illustrates the frequency of each subsequent action taken for each complication category.

**Figure 4:**
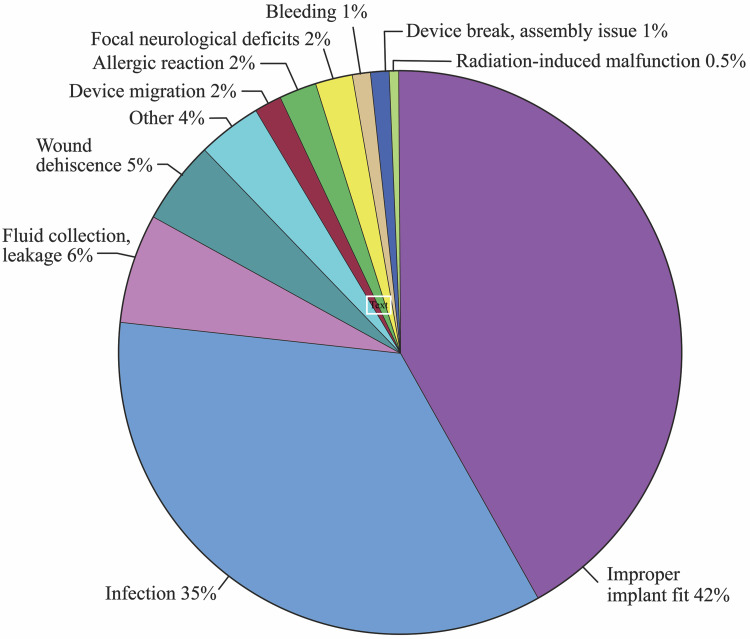
Complications associated with Synthes’ polyetheretherketone patient-specific implant

The Synthes (DePuy Synthes Companies, Massachusetts, United States) polyetheretherketone (PEEK) PSI had the most entries for complications of all reported brands (189 reports). The most frequent complication was improper implant fit (42%), followed by infection (35%), fluid collection or leakage (6%), and wound dehiscence (5%). Of the remaining complications, there were four reports due to focal neurological deficits, four cases of allergic reactions, three plates migrated, two plates broke, two procedures resulted in excessive bleeding, and one malfunctioned after exposure to radiation. There were seven other reports that did not specify the complication. The distribution of these complications is illustrated in Figure 4.

**Figure 5:**
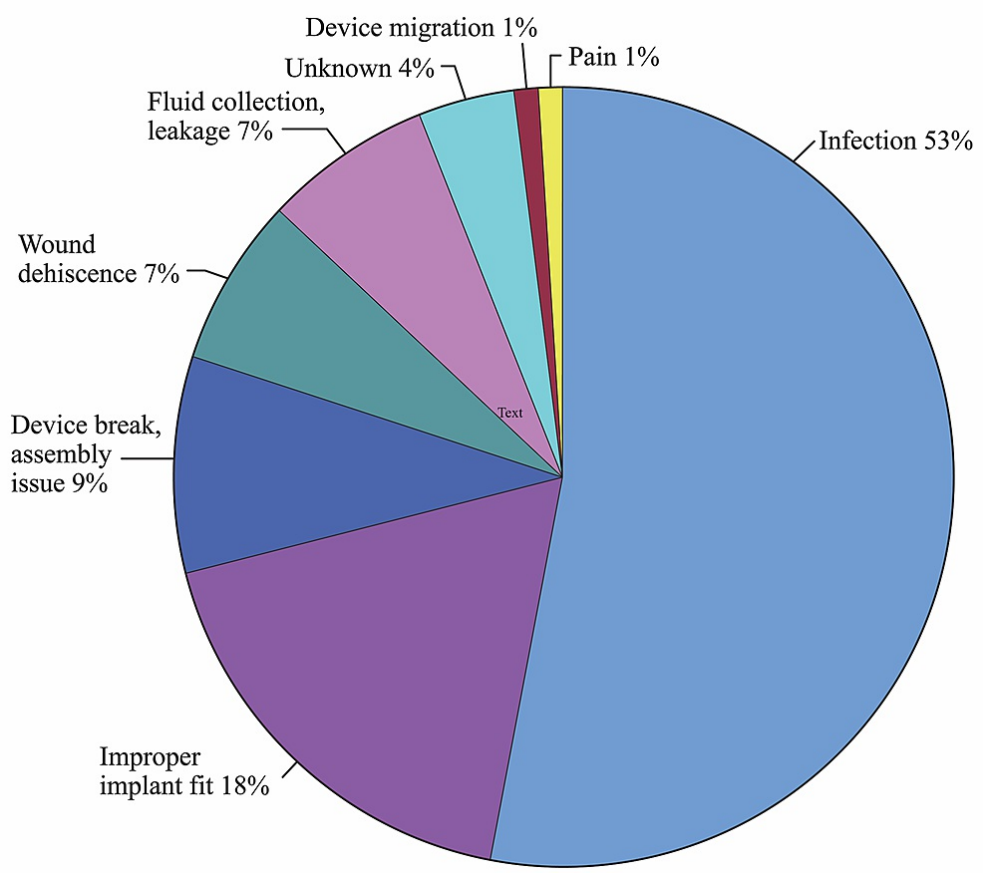
Complications associated with Biomet’s polyetherketoneketone patient-matched implant

Biomet’s (Zimmer Biome, Indiana, United States) hard tissue replacement (HTR) polyetherketoneketone (PEKK) patient-matched implant (PMI) was responsible for 19% of all complication reports. The most reported complication for the HTR-PEKK PMI was infection (53%). Improper implant fit (18%), device breaks and assembly issues (9%), wound dehiscence (7%), and fluid collection or leakage (7%) were less common complications for this brand and are illustrated in Figure 5. There was one case of device migration and one report of an implant causing significant pain to the patient. Three reports did not specify the complication that occurred and were regarded as unknown.

MedCAD’s (MedCAD, Dallas, USA) AccuShape polyetheretherketone (PEEK) PSI had 18 total complications reported. The most common complication for the AccuShape PEEK PSI was infection (50%). Wound dehiscence (17%) and improper implant fit (11%) were less common. There was one report for an allergic reaction and one report of an implant causing increased intracranial pressure. Unspecified complications accounted for 11% of reports.

Other brands, which were associated with complications, include KLS Martin’s (Tuttlingen, Germany) individual patient solutions (IPS) implants (15 reports), the Synthes' commercially pure (CP) titanium PSI (five), the OsteoMed (California, USA) OsteoMatch PMI (four), and the OssDsign Cranial PSI (one). There were 17 reports in which brands were unknown or not reported.

## Discussion

Although cranioplasty has a long recorded history of use following craniectomy, the challenge of selecting optimal implant materials for a given indication has recently regained much interest in the neurosurgical literature. The lack of a gold standard has created a diverse market of implant choices, resulting in many uncharacterized potential complications for various manufactures’ plates [3,11]. Thus, this discussion will focus on the results of complication and brand-specific data extrapolated from the MAUDE database by comparing this report’s findings to recently published literature.

When regarding speculating rates of complications in total, the authors reached out to the different cranioplasty manufacturers for a number of the total implants during this study’s timeframe. The representative for Biomet reported a total of 3161 implants during the study timeframe. MedCAD and Synthes were unable to provide a quote during this timeframe. This would mean that the complication rate would be approximately 2.0% for Biomet implants as an overall complication rate.

Infection is a known and frequently reported complication of cranioplasty [6,8,12-16]. This retrospective study found that infection was the most common complication reported overall. When Biomet’s HTR-PEKK PMI and MedCAD’s AccuShape PEEK PSI implants were used, infection was the most often reported complication (53% and 50%, respectively). Infection occurred in 35% of cases when the Synthes’ PEEK PSI was used. In a meta-analysis comparing outcomes of cranioplasty using PEEK, bone graft, and titanium implants, Punchak et al. reported an infection rate of 6% for PEEK implants [3]. Additionally, a multicentric review by Morselli et al. included a total of 233 patients receiving PEEK implants that were from various manufacturers. They reported an associated infection rate of 7.29%, of which 6.01% (82% of total) required a second surgery for treatment [6]. This study found that 93% of total infectious cases required subsequent surgery for management. This also is similar to the findings of this report as nearly 85% of infections required a return to the operating room.

One method of infection management proposed in the literature is creating adequate revascularization to ensure optimal uptake of antibiotics by tissues [13]. Zhang et al. also reported that adding anti-infective nanoparticles including silver nanoparticles, silk fibroin/gentamicin sulfate, and hydroxyfluorapatite to the surfaces of PEEK implants may help reduce infection rates [5]. These particles have been associated with increased bactericidal activity against both Gram-positive and Gram-negative bacterial infections [5]. Finally, reducing total surgical time has been associated with reduced infectious complications [12].

Patient-unique implants are a recent addition to available choices for cranioplasties [11]. These implants are created using computer-aided design and manufacture (CAD/CAM) based on patient's CT scan data and subsequently are 3D printed [5,9,11,12]. Synthes, Biomet, KLS Martin, OsteoMed, and OssDsign (Sweden) all report decreased operating times, better fits, and increased patient satisfaction with the use of PSI, but many of the MAUDE events report detailed intraoperative delays, in which the surgeon had to adjust the implant to achieve a better match to the patient’s defect [17-21]. Wolff et al. discuss the superiority of patient-unique implants and their use as nearly essential for the improvement in both functional and psychological states post-craniectomy, including the main benefit of PEEK implants as having excellent cosmesis [11]. However, our results showed 30% of complications overall due to incorrectly fitting implants. Synthes’ PEEK PSI was associated with improper implant fit in 42% of cases. Biomet’s HTR-PEKK PMI had an improperly fitting implant occurrence rate of 18%, while MedCAD’s AccuShape PEEK PSI had 11% of its complication reports due to improperly fitting implants. Although these rates are relative to one another and not absolute, future efforts should include increasing accuracy during the creation of PSI to decrease the time spent surgically revising the implant and increase patient satisfaction.

Breakage of plates was the third most common complication reported (6%, n = 21), of which 33% required replaced with new equipment. The use of Biomet’s HTR-PEKK PMI broke in seven cases. Synthes’ PEEK PSI broke in two cases, and there were no reported breaks for MedCAD’s AccuShape PEEK PSI. Four of the broken plate reports did not list an associated manufacturer. The breakage of plates used for cranioplasties has not been well documented in the literature at this time, but one retrospective study examining 156 cases of cranioplasty conducted by Sahoo et al. reported 14 cases of complications, of which two were due to plate breakdown [22]. This reflects only a 1% complication rate, but more research is needed before further conclusions can be drawn regarding cranioplasty plate breaks.

Wound dehiscence and poor healing following cranioplasty are important considerations because when present, wound dehiscence has been associated with a statistically significant increase in infections [23]. Overall, this study found that wound dehiscence and wound healing problems comprised 5% of all complications. The use of Synthes’ PEEK PSI was associated with wound dehiscence in 5% of cases. There were five reported cases of wound dehiscence when Biomet’s HTR-PEKK PMI was used (n = 77); three cases were reported for MedCAD’s AccuShape PEEK PSI (n = 18). A meta-analysis performed by Punchak et al. found that out of 183 cranioplasties in which PEEK implants were used, only two resulted in wound breakdown. A systematic review and meta-analysis performed by Liu et al. reported a statistically significant implant exposure rate of 2.3% for PEEK implants compared to Titanium-based plates (n = 128), supporting the notion that the PEEK implants have some benefit and potential for favorable outcomes [14].

Finally of note, there were three reported incidences of device exposure following radiation treatment. One plate was a KLS Martin IPS PEEK implant, which is reported to be resistant to gamma irradiation and magnetic resonance tomography (MRT) imaging [24]. The remaining reports were for Synthes’ PEEK PSI, which were not reported to be radiation-resistant [17]. A retrospective cohort study of 223 patients performed by Shalom and Gordon did not result in any post-radiation complications for cranioplasties when the implant material used was polymethyl methacrylate [25]. Conversely, titanium implants have been known to become exposed after post-treatment radiation [26,27]. More research is needed in this area to fully elucidate the effects of radiation post-cranioplasty for polyetheretherketone plates.

Limitations

The FDA cautions against using MDR data alone due to limitations including the potential for bias, inaccurate, incomplete, or unverified information contained within reports.

Individual healthcare workers are not mandatory reporters of complications; thus, the MAUDE database likely significantly under-reports complication rates. Therefore, the FDA cautions against use for prevalence management. The FDA encourages using information contained within MDRs in conjunction with other available resources for the evaluation of devices and subsequent treatment decisions.

Since the initial search for duplicates was performed manually, there could be errors in the correct identification of duplicate entries, so some reports could have been inadvertently included or left out. Adverse events that were not specified were not able to be classified into categories; thus, the reported totals could be lower than those in actuality. Complications reported in this review do not represent absolute rates and are relative to the others reported. Therefore, they should only be used for relative complication comparison and not for calculating prevalence rates.

Finally, pre-existing patient conditions that may have predisposed to complications, such as wound healing abnormalities, were not controlled for and therefore may inaccurately attribute complications to the implantable device.

## Conclusions

This study found that infections were the most frequent complication of cranioplasty overall with most cases resulting in implant removal. Other common complications included incorrectly sized implants and equipment breaks or assembly difficulties. DePuy Synthes’ PEEK PSI was associated with the majority of reported adverse events, and Biomet’s HTR-PEKK PMI accounted for most of the remaining event reports. The MAUDE database provides beneficial narrative descriptions for complications of cranioplasty allowing researchers to discern specific complication types of the implant. The database proves limited, however, in a way that individual healthcare workers are not mandatory reporters of complications. Thus, the MAUDE database likely significantly under-reports complication rates. Therefore, the FDA cautions against the use of prevalence management. In addition, there is also the potential for bias, inaccurate, incomplete, or unverified information contained within reports. Future research should be conducted to expand the knowledge and quantification of potential complications regarding cranioplasties.
